# The era of the digital natives is approaching: Insights into online peer-to-peer support for persons affected by prostate cancer

**DOI:** 10.1007/s00345-020-03114-1

**Published:** 2020-02-07

**Authors:** Markus W. Haun, Andreas Ihrig, Philipp Karschuck, Christian Thomas, Johannes Huber

**Affiliations:** 1 Department of General Internal Medicine and Psychosomatics, Heidelberg University, Im Neuenheimer Feld 410, 69120 Heidelberg, Germany; 2 Department of Urology, Medical Faculty Carl Gustav Carus, Technical University, Dresden, Germany

Health related virtual communities providing online peer-to-peer support (OPS) have gained great importance for patients’ advice and social support, because most patients want to participate more actively in treatment decision-making [1]. Recent meta-analyses of observational studies and RCTs have identified a positive effect of OPS in promoting changes in health-related behaviors [2, 3]. Although most of our patients are still so-called “digital immigrants”, the share of “digital natives” is rising throughout all disease entities. Patients who are currently online become more empowered, because virtual communities decrease the perceived stigma through their anonymity, allow patients to gather information, and enable patients who are restricted in their mobility to find peers [4]. While seeking OPS frequently improves the patients’ relationship with their healthcare professionals, there is evidence that healthcare providers still react negatively when patients disclose that they use OPS [5, 6]. Nevertheless, patients usually continue to use OPS while their need for feedback on the accuracy of information gathered online remains unaddressed. Indeed, it has recently been estimated that three in ten patients with localized prostate cancer (PC) revise their initial treatment decision based on their experience with OPS [7].

For persons affected by PC seeking OPS, a recent systemic review on 27 studies provides a comprehensive overview on the body of evidence concerning the role of OPS in treatment decision-making, quality of life, and potential caveats or adverse events [8]. Only one RCT with a small sample size of *n* = 40 was identified [9]. This study observed a short-term improvement of quality of life in the OPS group following a return to baseline at eight weeks. Observational and qualitative studies indicate that the exchange of information plays a greater role for patients with PC when compared to patients with breast cancer reflected in the finding that medical terms were the most common keywords. There is some evidence that OPS is also sought by caregivers, mainly to tackle with emotional distress. While certain risks like inaccurate health information and increased uncertainty were found, several cross-sectional studies underscored the great influence that OPS exerts on patients’ treatment decision-making.

Based on this synthesis of the current literature, what are the implications while the era of the digital natives is approaching? First, clinicians should be encouraged to accept that patients place much value on OPS and continue to do so, even when healthcare professionals oppose this need. In a time of rising numbers of digital natives, neglecting or dismissing the role of OPS is simply not an option anymore. Second, fully taking responsibility for a trusted patient-provider relationship, clinicians should therefore address patients’ need for discussing the accuracy of information gathered through OPS. In doing so, providers will not only account for the current body of evidence but also prepare the ground for a true shared decision-making process. Finally, with existing studies underscoring the feasibility of interventional trials in the context of OPS, clinical researchers should initiate well-designed and sufficiently powered RCTs accounting for patient and caregiver outcomes [10]. The worst-case scenario would be the expectable increase of OPS use without reliable evidence on its effects.
